# Modulation of Phase Separation by RNA: A Glimpse on N^6^-Methyladenosine Modification

**DOI:** 10.3389/fcell.2021.786454

**Published:** 2021-12-10

**Authors:** Yingfeng Su, Yasen Maimaitiyiming, Lingfang Wang, Xiaodong Cheng, Chih-Hung Hsu

**Affiliations:** ^1^ Women’s Hospital, Institute of Genetics, Department of Environmental Medicine, Zhejiang University School of Medicine, Hangzhou, China; ^2^ Department of Hematology of First Affiliated Hospital, Department of Public Health, Zhejiang University School of Medicine, Hangzhou, China; ^3^ Department of Obstetrics and Gynecology, Women’s Hospital, Zhejiang University School of Medicine, Hangzhou, China

**Keywords:** phase separation, N6-methyladenosine (m 6 A), biomolecular condensate, multivalence, RNA modification, RNA–RNA interaction, RNA–protein interaction

## Abstract

Phase separation is the driving force behind formation of various biomolecular condensates (BioMCs), which sub-compartmentalize certain cellular components in a membraneless manner to orchestrate numerous biological processes. Many BioMCs are composed of proteins and RNAs. While the features and functions of proteins are well studied, less attention was paid to the other essential component RNAs. Here, we describe how RNA contributes to the biogenesis, dissolution, and properties of BioMCs as a multivalence providing scaffold for proteins/RNA to undergo phase separation. Specifically, we focus on N^6^-methyladenosine (m^6^A), the most widely distributed dynamic post-transcriptional modification, which would change the charge, conformation, and RNA-binding protein (RBP) anchoring of modified RNA. m^6^A RNA-modulated phase separation is a new perspective to illustrate m^6^A-mediated various biological processes. We summarize m^6^A main functions as “beacon” to recruit reader proteins and “structural switcher” to alter RNA–protein and RNA–RNA interactions to modulate phase separation and regulate the related biological processes.

## Introduction

Compartmentalization is a common strategy of cells to ensure timely and spatial execution and coordination of various biochemical reactions. While many compartments called membrane-bound organelles are surrounded by phospholipid bilayers, membraneless organelles, biomolecular condensates (BioMCs) lacking lipid bilayers, also constitute another form of cellular compartments. Although BioMCs and membrane-bound organelles are both efficient to accomplish biochemical reactions within the organelles, they differ significantly in their biogenesis, component, sensitivity to the environment, and so on (Aguilera-Gomez and Rabouille, 2017). In 2009, P granules, a type of protein-rich BioMCs essential for zygogenesis in *Caenorhabditis elegans*, were found to exhibit gel-like behaviors (Brangwynne et al., 2009). Since then, phase separation, especially liquid–liquid phase separation (LLPS), has gained broad attention as a physicochemical mechanism for forming both nuclear and cytoplasmic membraneless structures. To date, many distinct BioMCs are reportedly driven by phase separation, including stress granules (SGs) (Molliex et al., 2015; Guillén-Boixet et al., 2020; Sanders et al., 2020; Yang et al., 2020), processing bodies (PBs) (Smith et al., 2016), spindle apparatus (Jiang et al., 2015), and centrosome (Woodruff et al., 2017) in the cytoplasm along with nucleolus (Brangwynne et al., 2011; Feric et al., 2016; Yao et al., 2019) and paraspeckles (Hennig et al., 2015; West et al., 2016; Yamazaki et al., 2018) in the nucleus. In addition to conventional condensates, burgeoning BioMCs participating in gene expression such as heterochromatin (Strom et al., 2017), super enhancer (Hnisz et al., 2017; Sabari et al., 2018), and mediator complex (Boija et al., 2018; Guo et al., 2019) are all formed and modulated by phase separation.

Based on the current literature, phase separation is elicited by multivalent low-affinity interactions, which usually happen among protein–protein, protein–RNA, and RNA–RNA (Banani et al., 2016). Through phase separation, protein and/or RNA components concentrate to form “droplets” distinct from surrounding dilute phase, which exhibit unique properties such as spherical shape and rapid dynamics (Banani et al., 2017; Alberti et al., 2019), thereby exerting various functions. Apart from well-known membraneless cellular compartments (e.g., SGs and PBs) functioning as important organelles *via* phase separation, it was reported recently that phase separation might associate with oncogenic fusion protein degradation by heat stress (Maimaitiyiming et al., 2021). The fluidity of BioMCs allows them to organize dynamically and function efficiently. Inversely, the arrest of BioMCs’ dynamics is correlated with some pathological processes (Mathieu et al., 2020). Take TDP-43 as an example, abnormal nuclear shuttle and decreased nuclear pore complex caused by persistent stress or cell aging could lead to the accumulation of TDP-43 in the cytoplasm; the abnormal TDP-43 accumulation results in decreased dynamics of phase-separated TDP-43 droplets and converts the droplets into gel or solid aggregations, which could induce neurotoxicity (Gasset-Rosa et al., 2019). Furthermore, the decreased RNA-binding capacity of TDP-43 induced by mutation in RNA-recognition motif also exhibits reduced dynamics and promotes similar pathological progression (Mann et al., 2019). Therefore, it is worth to further study the mechanism by which BioMCs assemble and function, so as to exploit ways to modulate this physiological process for developing novel treatment approaches for diseases caused by abnormal phase separation.

Many BioMCs are ribonucleoprotein (RNP) granules containing RNA and RNA-binding proteins (RBPs) (Banani et al., 2016; Banani et al., 2017), and the heterogeneity of composition may dictate the heterogeneity of function (Banani et al., 2016). The majority of the available literatures focus on the contribution of proteins to phase separation. By performing targeted mutagenesis, many studies have demonstrated that low-complexity domains (LCDs)/intrinsically disordered regions (IDRs) in RBPs contribute to multivalence and are essential in RNP granule formation (Boeynaems et al., 2018). However, in sharp contrast to the role of RBPs in phase separation, much less attention was paid to RNA. Notably, IDRs of some RBPs provide structural flexibility to make adequate contact with their partner RNAs (Molliex et al., 2015; Basu and Bahadur, 2016). In addition, RNA-binding domains (RBDs) in RBPs are required for BioMCs’ assembly, while IDRs are dispensable in certain cases (Guillén-Boixet et al., 2020; Sanders et al., 2020; Yang et al., 2020). Consistent with these findings, several studies showed that the addition of RNA lowers the concentration threshold for RBPs to trigger phase separation (Gao et al., 2019; Ries et al., 2019; Guillén-Boixet et al., 2020; Wang et al., 2020; Yang et al., 2020). These findings indicate that RNA plays a role in phase separation, at least by interacting with RBPs. Furthermore, protein-free total RNA extracted from yeast self-assembles into droplets (van Treeck et al., 2018), implying RNA–RNA interaction potentially contributes to phase separation as well.

Here, we mainly focus on the contribution of RNA in phase separation. Given post-transcriptional RNA modification is widely distributed and of critical importance to RNA processing and function (Zaccara et al., 2019), we summarize current studies on how RNA modification, especially N^6^-methyladenosine (m^6^A), contributes to phase separation and discuss its potential biological significance.

## RNA Modulates the Formation and Properties of Biomolecular Condensates by Regulating Phase Separation

The interaction between biological macromolecules is the core event in phase separation, and the valence as well as affinity of the interaction are the key parameters for regulating phase separation (Tauber et al., 2020a). Previously, the role of proteins in phase separation has been widely reported. IDRs are rich in disorder-promoting amino acids (such as Q, S, N, Y, and G) and prone to form pi–pi, cation–pi, or bipolar interactions. Thus, proteins containing IDRs are generally involved in phase separation through providing multivalent interactions (Oldfield and Dunker, 2014). Accruing evidence indicated that RNA, as a flexible and variable molecule with the properties to interact with multiple partners including protein and RNA (Roden and Gladfelter, 2021), is a potentially powerful multivalency provider in phase separation. In this part, we discuss how RNA affects the assembly and properties of BioMCs through modulating phase separation.

### RNA: An Innate Multivalence Provider

**FIGURE 1:**
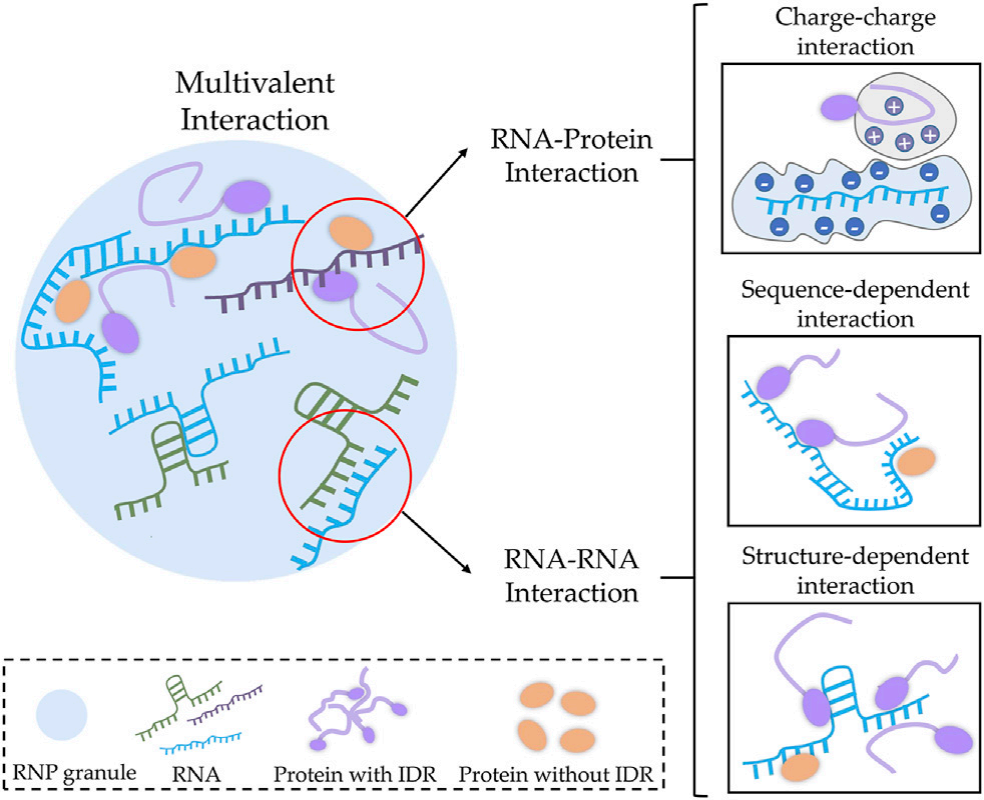
RNA regulates biomolecular condensates’ (BioMCs) properties through providing multivalence. RNA acts as an important multivalence provider through interacting with other bio-macromolecules (such as proteins and RNA) *via* charge–charge, sequence-specific, and structure-dependent interactions.

On one hand, RNA provides multivalency by introducing a non-specific negative charge to modulate phase separation (Figure 1). Most of the interactions that modulate phase separation are electrostatic in nature. To a certain extent, polymers with opposite charges can promote the condensation of biological macromolecules. For instance, during RNP granule formation, LCDs of protein components can drive phase separation through non-covalent charged interaction (Wang et al., 2018), and this type of interaction occurs either between LCDs or between LCDs and other domains of proteins (Monahan et al., 2017; Qamar et al., 2018). Similarly, adding cationic spermine to the solution promotes the condensation of negatively charged poly-U RNA into small droplets, which exhibit fluidity and temperature sensitivity. This phase-separated system reaches its highest turbidity at a specific ratio of positive and negative charges (Aumiller et al., 2016). It indicates that the negative charge of RNA favors phase separation once oppositely charged substances are added to the system by providing multivalency for the charge–charge interaction.

Unlike DNA, most cellular RNAs are single-stranded, leading to complete exposure of phosphate backbones and bases to the surrounding environment. This is beneficial for establishing an interaction between RNA and positively charged molecules. In a model composed of poly-U RNAs and cationic peptides, dephosphorylation at a serine is sufficient to cause a charge–charge interaction between the negatively charged RNA and the positively charged peptides, thereby mediating phase separation. Conversely, phosphorylation at this serine reverses this process, causing the formed droplets to dissolve (Aumiller and Keating, 2016). In addition, some protein domains rich in positive charges, such as the arginine/glycine-rich (RGG) domain, are believed to bind to negatively charged RNA non-specifically to promote protein–RNA interaction (Elbaum-Garfinkle et al., 2015; Lin et al., 2015; Saha et al., 2016; Yoshizawa et al., 2018), which displays the potential of RNA to influence phase separation through charge–charge interaction. It is worth noting that although the addition of oppositely charged biomolecules initially promotes droplet assembly through mechanisms including charge–charge interactions, excess amount of spermine will cause dissolution of small droplets formed by poly-U RNA (Aumiller et al., 2016), and superabundant RNA in the phase-separated system will trigger a charge inversion and disassembly of RNP. This phenomenon is called reentrant phase transition (Banerjee et al., 2017). Collectively, RNA can provide negative charge to regulate the generation and depolymerization of BioMCs in a sequence-independent manner.

On the other hand, RNA also provides protein or RNA binding sites in a sequence- and structure-dependent manner to enhance RNA–protein or RNA–RNA interactions (Figure 1). RNA structure is relatively flexible, allowing it to either maintain an unstructured state and expose the combination motif for other molecules or fold into complicated structures such as hairpin, helical regions, tetra loops, G-quadruplexes, etc. (Miao and Westhof, 2017). The structural diversity allows RNA to form multiple conformations. Although the RNA structure is thought to play a role to limit access of RBPs to its target specific RNA motifs, certain non-specific RBPs bind to RNA *via* recognizing RNA structure (Jankowsky and Harris, 2015). For instance, the Whi3 protein preferentially binds to stem loops formed by mRNAs such as *CLN3*, *BNI1*, and *SPA2* and regulates secondary structures of these mRNAs (Langdon et al., 2018). Collectively, RNA can interact with other molecules in various ways including non-specific charge–charge, sequence-dependent, and structure-dependent manners, which increase the probability to form RNA-dependent massive complexes, thereby promoting phase separation. Interestingly, numerous *in vitro* experiments have shown that adding RNA to the protein solution could result in the reduction of protein concentration through forming phase-separated droplets (van Treeck and Parker, 2018; Tauber et al., 2020a), but other studies showed that excessive or high-affinity RNAs prevent phase separation by competitively inhibiting protein–protein interaction (Maharana et al., 2018; Gasset-Rosa et al., 2019; Mann et al., 2019), suggesting the ratio of the amount between RNA and protein is a critical parameter for phase separation. Notably, longer RNAs are preferred to promote the formation of phase-separated structures as they might possess multiple sites to interact with other partners. Proving this notion, in some phase-separated intracellular granules, such as P-granule (Saha et al., 2016) and stress granule (Khong et al., 2017), there is a preference for enrichment of longer RNAs. Together, these findings suggest that RNA plays an important role in phase separation through modulating RNA–protein interaction.

RNA–RNA interaction can occur in a variety of ways including Watson–Crick base-pairing, non-Watson–Crick interaction, and base stacking. For two random long RNAs, these kinds of interactions are potentially widespread (van Treeck and Parker, 2018). Longer length, higher GC content, and binding with RBPs are favorable for this kind of interaction, while a structured and translated state inhibits it (van Treeck and Parker, 2018; Roden and Gladfelter, 2021). Multiple RNAs self-assemble *in vitro* independent of RBPs (Aumiller et al., 2016; Langdon et al., 2018; Boeynaems et al., 2019). For instance, the protein-free total RNA extracted from yeast undergoes self-assembly in an environment mimicking physiological state, implying that RNA–RNA interaction alone is sufficient to mediate phase separation (van Treeck et al., 2018). The formation of paraspeckles is also reportedly mediated by RNA–RNA interaction (Chujo and Hirose, 2017). Likewise, Barr body (Cerase et al., 2019), a well-studied BioMCs that potentially assembles through phase separation, exhibited intermolecular interactions among *XIST*s (Lu et al., 2016). Other studies also showed the importance of RNA–RNA interaction in BioMC formation *via* modulating phase separation. Ras-GAP SH3 domain-binding proteins (G3BPs) are important assembly factors of SGs, and knockout of G3BPs disrupts SGs’ formation (Guillén-Boixet et al., 2020; Yang et al., 2020; Hofmann et al., 2021); Tauber et al. (2020b) found that promoting RNA–RNA interaction by inhibiting eIF4A, a disruptive protein for RNA–RNA interaction, results in SG reformation in G3BPs knockout cells.

Notably, similar to protein aggregation diseases, short nucleotide repeats containing RNAs undergo sol-gel transition by multivalent base-pairing (Gasset-Rosa et al., 2019), and inhibition of aberrant RNA–RNA interaction by adding monomeric RBP leads to disassembly of RNA droplets (Sheth and Parker, 2003; Chujo and Hirose, 2017). As a whole, the complicated network structure formed by RNA–RNA interactions might directly trigger phase separation or provide platforms for proteins to condensate and undergo phase separation.

### The Role of RNA in Phase Separation: Driver, Regulator, and Buster

As an important biomacromolecule with relatively large size, complicated sequence, and flexible structure, RNA serves as a scaffold for the interaction between biomacromolecules including RNA–protein and RNA–RNA, to achieve multivalence and modulate phase separation. Specifically, RNA regulates the valence and affinity of interaction with other biomacromolecules, playing different roles in phase separation. First, an increase of regional concentration of specific RNAs is the driver of local BioMC formation. In the nucleus, this is often mediated by transcription activities, which is in synergy with the establishment of some nuclear structures. For example, paraspeckles are formed at the local transcription site of lncRNA *NEAT1* (Chujo and Hirose, 2017), and pre-rRNA transcription is involved in the formation as well as maintenance of nucleolus (Brangwynne et al., 2011; Feric et al., 2016; Yao et al., 2019). In the cytoplasm, the formation of SGs and PBs is dependent on the increase of the pool of untranslated naked RNAs. Inhibiting RNA degradation or blocking the initiation of translation at the overall level promotes the biogenesis of SGs and PBs (Sheth and Parker, 2003; Mazroui et al., 2006). On the contrary, degrading mRNA or trapping them on the ribosomes inhibits the biogenesis of SGs (Khong and Parker, 2018; Burke et al., 2019; Guillén-Boixet et al., 2020; Yang et al., 2020), suggesting the indispensable role of RNAs in the formation of BioMCs.

In addition to acting as seeds and platforms for formation of RNP granules, RNA also serves as a regulator for the properties, such as dynamics and subcompartmentalization, of RNP granules (Tauber et al., 2020a; Trcek et al., 2020). Generally, higher valency among biomacromolecules represents a slower exchange rate (Banani et al., 2016), and this provides an explanation for why RNA leads to slower dynamics in RNP granule both *in vitro* and *in vivo* (Banani et al., 2016; Tauber et al., 2020a). In physiological conditions, specific RNAs determine the liquid-like dynamics of specific RNP granules and create immiscible granules (Zhang et al., 2015; Langdon et al., 2018). In repeat expansion diseases, RNA foci exhibited less dynamics and gelation in a repeat length-dependent manner (Fay et al., 2017; Jain and Vale, 2017). For specific RBPs, higher affinity of RNAs induces less dynamics, indicating a lower off-rate from RNAs (Tauber et al., 2020a). This was proved by domain-swapping experiments of G3BP1 (Yang et al., 2020). Specifically, adding more RBDs (KH domain; ZnF domain; G3BP1 RBD) resulted in less dynamics of G3BP1. The internal substructures of RNP granules remain largely unknown (Feric et al., 2016). Recently, Trcek et al. (2020) showed that mRNAs could self-assemble into homotypic assemblies within granules, and the regulation of spatial organization is due to sequence but not general RNA–RNA interactions.

Contrary to acting as “seeds” or “glue,” when the concentration of RNA is excessively high, it exhibits a destructive effect on the already formed BioMCs. This effect may be caused by charge inversion due to the introduction of massive negatively charged RNA or hindering of protein–protein interaction by excess RNA (Banerjee et al., 2017; Mann et al., 2019). Given that the concentration of RNA in the nucleus is more than 10 times higher than that of the cytoplasm (Maharana et al., 2018), RNA in the nucleus may serve as buffer to limit the abnormal aggregation of FUS, TDP43, hnRNPA1, and other nuclear proteins (Maharana et al., 2018; Mann et al., 2019). On the contrary, under senescence or repeated external stimuli, TDP43 abnormally locate and accumulate in the cytoplasm to produce pathological aggregation (Gasset-Rosa et al., 2019). Notably, a dynamic RNA/protein ratio change may play a role in controlling RNP granule with tunable lifetimes through either promoting or preventing phase separation (Banerjee et al., 2017; Henninger et al., 2021). For instance, Henninger et al. (2021) reported that newborn RNAs contribute to feedback control of transcriptional condensates by reentrant phase transition. Specifically, small non-coding RNAs produced at the initiation of transcription promote the formation of transcription condensates mediated by phase separation, but the large amount of long-strand RNA produced by transcription elongation will in turn promote the dispersion of the transcription condensates, by which newborn RNA completes feedback regulation. Collectively, RNA plays various roles during phase separation depending on the context.

### RNA m^6^A Modification Modulates Phase Separation

As mentioned above, the multivalent interaction is the key event for both formation and modulation of phase separation-mediated BioMCs. Recent studies showed that the interaction is widely affected by post-translational modification (PTM) of proteins participating in phase separation (Hofweber and Dormann, 2019). For instance, arginine methylation at RGG regions in LCDs reduces the phase separation potential of hnRNPA2 by disrupting arginine-mediated arginine–aromatic interactions (Ryan et al., 2018). Delocalized pi system can be provided by both aromatic amino acids and nucleobases, implying RNA–protein interaction is also a potential target modulated by arginine methylation. Proving this notion, arginine demethylation at the RGG region of G3BP1, which is considered important for RNA binding and initiation of SG establishment (3–6), is prerequisite for SG assembly (Tsai et al., 2016).

Similar to post-translational modifications of proteins, RNA is subject to numerous post-transcriptional modifications (over 160), which play critical roles in modulating the properties of RNA and regulating RNA metabolism (Machnicka et al., 2013). Unlike 5-methylcytosine (m^5^C) on the CpG island of DNA (Roels et al., 2020), m^6^A is the most abundant and well-studied modification on eukaryotic mRNA. m^6^A is a dynamic and reversible modification regulated by three groups of “m^6^A modifiers,” including “m^6^A writers” (m^6^A methyltransferases), “m^6^A readers” (m^6^A-binding proteins), and “m^6^A erasers” (m^6^A demethylases) (Zaccara et al., 2019; Liang et al., 2020). The m^6^A writers (METTL3/METTL14/WATP complex, METTL16, etc.) catalyze m^6^A in a site- and transcript-specific manner, and the m^6^A erasers (FTO and ALKBH5) specifically remove the methyl group. These enzymes make m^6^A modification reversible and adjustable. Notably, the function of m^6^A modification is extensively decided by its readers (YTH domain containing proteins, IGF2BPs, HNRNPs, etc.). Each of these readers exhibits distinct function in regulating the fate of m^6^A-modified RNA. For instance, YTHDC1 regulates alternative splicing (Xiao et al., 2016) and nuclear export (Roundtree et al., 2017), YTHDC2 promotes translation initiation (Hsu et al., 2017) and RNA degradation (Wojtas et al., 2017), YTHDF1 enhances the translation (Wang et al., 2015), YTHDF2 promotes RNA degradation (Wang et al., 2013), and YTHDF3 exhibits similar functions with both YTHDF1 and YTHDF2 depending on the context (Shi et al., 2017). Therefore, m^6^A modification is involved in the elaborate regulation of many bioprocesses including cellular stress responses (Zhou et al., 2015; Xiang et al., 2017; Ji et al., 2021; Ninomiya et al., 2021), tumorigenesis (Deng et al., 2018; Liang et al., 2020; Rosselló-Tortella et al., 2020), and differentiation (Edens et al., 2019; Song et al., 2019).

Recently, several groups have reported that multivalent m^6^A-modified RNAs act as scaffolds to gather YTHDF proteins and thus lead to phase separation both *in vitro* and *in vivo* (Gao et al., 2019; Ries et al., 2019; Fu and Zhuang, 2020; Wang et al., 2020). These m^6^A-modified mRNA–YTHDF protein complexes are subsequently partitioned into SGs and potentially influence the fate of m^6^A-containing mRNA stored in SGs (Gao et al., 2019; Ries et al., 2019; Fu and Zhuang, 2020). These studies reveal the strong potential of m^6^A-modified RNA over regulating phase separation. However, it remains elusive what molecular mechanisms are exploited by m^6^A modification other than multivalently recruiting YTHDF proteins to influence phase separation and regulate multiple bioprocesses.

In this part, based on the current literature, we describe how m^6^A modification alters the properties of target RNA (including RNA conformation, the capacity for protein-binding, and the affinity to interact with other RNAs) and further discuss how these changes potentially contribute to phase separation.

### m^6^A Acts as a “Beacon” to Recruit Reader Proteins

Interacting with diverse m^6^A readers is recognized as a major mechanism for regulating various fate for m^6^A-harboring RNA. By now, a large group of RBPs have been verified to directly bind to m^6^A-modified RNA (Zaccara et al., 2019). Among them, YTH domain-harboring proteins are the first group of readers to be discovered in an m^6^A pull-down assay (Dominissini et al., 2012). Structural studies revealed that m^6^A resides in a deep cleft formed by three hydrophobic residues in YTH domain–m^6^A-modified RNA complex, and the methyl–pi interaction between the methyl group of m^6^A and the rings of the two tryptophan residues constitutes the basis of the preference of YTH domain toward m^6^A modification (Liao et al., 2018). The members of cytosolic YTHDF family (YTHDF1/2/3) share high similarity in length, amino acid composition, and conformation; except for C-terminal YTH domain of around 15 kDa, YTHDF family proteins also contain an around 40-kDa LCD including prion-like domain (Patil et al., 2018). This structural feature implies potential for YTHDFs to be involved in phase separation. Indeed, the LCDs of all three YTHDFs are sufficient to trigger phase separation in a concentration-dependent manner without RNA *in vitro* (Gao et al., 2019); notably, glutamine (Q)-rich domain is important for the capacity to undergo phase separation, as changing all Q to alanine (A) in this region led to loss of phase separation potential (Wang et al., 2020). Consistently, the YTH domain alone failed to undergo phase separation even at a high concentration (Ries et al., 2019). Although the YTH domain is not required for phase separation *in vitro*, it plays an important role in phase separation of YTHDFs through binding to m^6^A-modified RNA. The addition of m^6^A-modified RNA can lower the concentration threshold needed to form YTHDF condensates in an m^6^A valency-dependent manner (Gao et al., 2019; Ries et al., 2019; Wang et al., 2020), but the enhancing effect of multivalence by m^6^A-modified RNA disappears when the m^6^A-binding capability of YTHDFs is compromised either by mutation or deletion of YTH domain (Gao et al., 2019; Wang et al., 2020). These studies suggest that multivalent m^6^A-modified RNA may act as scaffolds to concentrate YTHDFs in a small area leading to phase separation.

**FIGURE 2:**
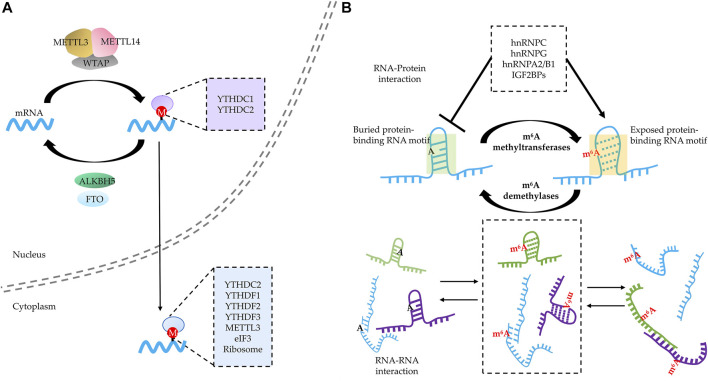
RNA N^6^-methyladenosine (m^6^A) modification regulates RNA–RNA and RNA–protein interactions of modified RNA. **(A)** RNA m^6^A modification acts as a “beacon” to directly recruit various m^6^A readers in both the nucleus and cytoplasm. **(B)** RNA m^6^A modification modulates RNA–RNA and RNA–protein interaction through “structural switcher” function. m^6^A modification promotes the instability of RNA base complementary pairing and thus leads to deconstruction of the corresponding structure, reshaping the spectrum of RNA–protein and RNA–RNA interaction.

More importantly, the phenomena observed *in vitro* may have an important implication *in vivo* given the tight correlation between m^6^A-modified RNA–YTHDFs complex and biomolecular condensates including SGs, PBs, and neuronal RNA granules (Molliex et al., 2015; Gao et al., 2019; Ries et al., 2019; Fu and Zhuang, 2020; Patil et al., 2018). First, m^6^A-modified RNA–YTHDFs complex colocalized with SGs formed by overexpressing G3BP1 or various stresses including oxidative stress, heat shock, and ER stress (Gao et al., 2019; Ries et al., 2019; Fu and Zhuang, 2020). Second, excluding the interference of length, which is an important parameter for RNA targeting into biomolecular condensates (van Treeck et al., 2018), the SGs mediated by various stresses consistently show a preference for m^6^A-modified mRNA in a valency- and stoichiometry-dependent manner (Wang et al., 2020). In addition to SGs, PBs also exhibit the preference in a stoichiometry-dependent manner regardless of the length of mRNA (Wang et al., 2020). Third, the capability of m^6^A binding is essential for YTHDFs to partition into BioMCs *in vivo*. For instance, METTL14 knockout reduced targeting of YTHDF2 to PBs under normal conditions and markedly reduced the relocation of YTHDF2 into SGs under stress. Similarly, the compromised m^6^A-binding capacity resulting from introducing mutation to YTH domain lowered YTHDF2 content in SGs as well (Ries et al., 2019). For YTHDF1 and 3, knockdown of any or both of them disturbed SG formation, and YTHDF protein expression was able to restore SGs, while the overexpression of YTH domain-deficient truncation failed to do so. Consistently, by overexpressing a dominant-negative YTHDF1 to compete m^6^A binding with endogenous wild-type YTHDFs, SG formation was partially impaired under stress conditions (Fu and Zhuang, 2020). It is worth noting that when N-IDR was swapped with CRY2olig domain, which can undergo blue light-induced oligomerization (Taslimi et al., 2014), blue light succeeded to oligomerize recombinant YTHDF1 but failed to induce SG assembly in YTHDF1/3 KD cells, even under stress conditions (Fu and Zhuang, 2020). These findings suggest that neither oligomerization of YTHDFs nor YTHDF interaction with other proteins is sufficient for phase separation and SG assembly. On the other hand, it is evident that m^6^A acts as a “beacon” to recruit readers (Figure 2A) and mRNA harboring multivalent m^6^A modification and serves as scaffolds to gather multiple reader proteins, which may enhance phase separation and modulate SGs. However, although YTHDF1/3 knockdown largely reduces SG formation, SG assembly seems not fully dependent on m^6^A modification (Fu and Zhuang, 2020). A recent study showed that SGs assemble from the summation of a multitude of RNA–protein, RNA–RNA, and protein–protein interactions rather than only one of them (Matheny et al., 2021), and this explains why the length of mRNAs as well as the number of potential interactions would play a major role in the formation of condensates (Khong et al., 2017). Given the different conditions applied and heterogeneity of SG assembly mechanism (van Leeuwen and Rabouille, 2019), both m^6^A modification and YTHDF proteins contribute to phase separation of m^6^A RNA–YTHDF complex.

Another member of YTH domain-harboring protein with the potential to undergo phase separation is YTHDC1, which mainly localizes in the nucleus and also contains a large LCD similar to YTHDFs (Patil et al., 2016). YTHDC1 was first found to localize in dot-like subnuclear condensates named “YT bodies” (recognized as nuclear speckles now), a membraneless structure that exhibited dynamics and is subjected to regulation by transcription state (Nayler et al., 2000). The function of YTHDC1 is tightly correlated with nuclear bioprocesses that are deemed to be driven or modulated by phase separation. First, YTHDC1 may participate in remodeling chromatin structure and gene silencing mediated by heterochromatin through phase separation (Peng et al., 2020). To be specific, nascent RNA with m^6^A sites recruits KDM3B, one of the histone demethylases, through the scaffolding role of YTHDC1 and thus decreases the H3K9me2 levels to potentially participate in chromosome remodeling (Li et al., 2020a). In addition, YTHDC1 also binds to transcripts of retrotransposons in an m^6^A-dependent manner in mouse ESCs and further catalyzes H3K9me3 modification of target chromosome by recruiting SETDB1 and NCL–KAP1 complex (Chen et al., 2021a; Liu et al., 2021). Specifically, *LINE1*, a lncRNA with multiple m^6^A peaks and YTHDC1 binding sites, forms the *LINE1*–NCL–KAP1complex, which plays a role in H3K9me3 installation and thereby modulates the expression of two cell state-related retrotransposons. Another evidence to prove this notion is Barr body, a lncRNA *XIST*–protein complex inducing heterochromatinization of the X chromosome, which is speculated to be mediated by phase separation (Cerase et al., 2019). YTHDC1 preferentially recognizes m^6^A residues on *XIST* and further recruits repressive proteins to chromatin to achieve gene silencing. It is worth noting that *XIST* harbors more than 70 m^6^A sites, implying a strong potential for scaffolding m^6^A readers (Patil et al., 2016). Second, YTHDC1 is able to reshape nuclear speckles and modulate transcription. Another lncRNA *MALAT1* with m^6^A modification acted as scaffold to recruit YTHDC1 to nuclear speckles, and the YTHDC1 anchoring played an important role in maintaining the composition and genomic binding sites of nuclear speckles. Similar to that of lncRNA *XIST* (Patil et al., 2016), as many as 31 high-confidence *MALAT1*–m^6^A motifs were identified (Wang et al., 2021). Third, YTHDC1 participates in alternative splicing, which is also potentially modulated by phase separation (Peng et al., 2020; Ninomiya et al., 2021). YTHDC1 may shortly bind to methylated nascent RNA and further stabilize SRSF3 (and displace SRSF10) to promote exon inclusion, and the LCD in the C-terminus is important for its interaction with SRSF3/10 (Xiao et al., 2016), suggesting that this is probably a phase separation-dependent phenomenon. Ninomiya et al. (2021) found that YTHDC1 as well as m^6^A–writer complex components could be sequestered inside nuclear stress bodies by binding to the m^6^A-modified lncRNA HSATIII, thereby repressing the m^6^A-dependent splicing of pre-mRNAs in the nucleoplasm. Recently, Cheng et al. (2021) have proven that m^6^A-modified mRNA and YTHDC1 can form m^6^A–YTHDC1 condensates in a phase separation-dependent manner, and this condensate in acute myeloid leukemia cells may protect some mRNA of malignance from degradation.

As a whole, several m^6^A readers themselves have a potential to trigger phase separation, and their anchoring on m^6^A-modified RNA strongly enhances this potential.

### m^6^A Acts as a “Structural Switcher” to Modulate the Spectrum of RNA–Protein and RNA–RNA Interaction

Liu et al. (2015) demonstrated that m^6^A acts as a “structural switcher” to change the conformation of RNA harboring m^6^A modification. Although m^6^A modification could not preclude the Watson–Crick base pairing between A and U, it induces the methylamino group rotating from energetically favored *syn* geometry on the Watson–Crick face to higher-energy anti-conformation, thus destabilizing the RNA duplex. The notion of m^6^A destabilizing base pairing was further verified by the kinetic research that showed introducing m^6^A significantly lowers the rate of duplex annealing, providing support for how m^6^A reshapes the kinetics of conformational transition toward single-string preference (Shi et al., 2019). On the other hand, at the unstructured region, m^6^A modification stabilizes the conformation due to stronger base–stacking interaction (Roost et al., 2015). Likewise, an *in vivo* transcriptome-wide RNA structure mapping study presented direct structural evidence that m^6^A affects RNA structure, favoring the transition from paired to unpaired RNA (Spitale et al., 2015).

By altering the RNA structure, m^6^A modification would help to recruit the RBPs that prefer to bind linear, unfolded RNAs (Figure 2B). In fact, some RBPs tend to bind to m^6^A-modified RNA because of the “structural switcher” function of m^6^A. Among them, several members of the heterogeneous nuclear ribonucleoprotein (HNRNP) family are well-studied (Liu et al., 2015; Zhou et al., 2016; Liu et al., 2017; Wu et al., 2018). For instance, HNRNP C recognizes the U-tract which are often buried by A-tract at the stem structure. The m^6^A modification of an A on the A-tract is capable to destabilize the region where the U-tract is located and increases the accessibility of the U-tract to HNRNP C (Liu et al., 2015; Zhou et al., 2016). Except for unmasking the target complementary sequences, m^6^A modification is capable of increasing the accessibility of its located region as well. HNRNP G binds to a purine-rich motif that includes the m^6^A site, and the m^6^A modification helps altering the structure to increase motif accessibility (Liu et al., 2017). Of note, HNRNP G binds to m^6^A-modified RNA through its LCD, which is able to undergo self-assembly (Liu et al., 2017); this suggests that the m^6^A modification leads to the “partner switch” of HNRNP G from protein to RNA. Another member of HNRNPs regarded to bind to m^6^A-mediated structural switch RNA is HNRNPA2/B1, which was revealed by structural, biochemical, and bioinformatics studies (Liao et al., 2018; Wu et al., 2018). Apart from HNRNPs, another group of m^6^A readers that may also bind to different targets in a structural switch-dependent manner are IGF2BPs (Sun et al., 2019), which were reported to enhance the stability and translation of m^6^A-modified mRNA (Huang et al., 2018). Therefore, in addition to recruit m^6^A readers directly binding to m^6^A sites, m^6^A-mediated structural switch of RNA contributes to binding multivalence for m^6^A-modified RNAs as well, which participates in the regulation of phase separation.

Of note, m^6^A does not consistently promote RNA–protein interactions. m^6^A also showed an ability to repress RBP binding; for instance, m^6^A modification may impede the formation of RNA structures needed for HUR binding (Wang et al., 2014). Several other “anti-readers” were verified by high-throughput screening (Edupuganti et al., 2017; Sun et al., 2019), such as LIN28A, EWSR1, G3BP1, and G3BP2, all of which were displaced when m^6^A appear in their binding sites. By recruiting and repelling RBPs, m^6^A potentially changes the spectrum of the RNA–protein interaction, which would contribute to the dynamic modulation of phase separation.

Another role for molecular switch mediated by m^6^A is to regulate the kinetics of RNA–RNA interactions (Figure 2B). As mentioned above, the addition of m^6^A may promote the dissolution of local duplex (e.g., steam, etc.) and tend to induce linear, unstructured conformation, which would accelerate the formation of trans-RNA–RNA interaction (van Treeck and Parker, 2018; Roden and Gladfelter, 2021). Therefore, m^6^A modification potentially serves as a kinetic regulator to reshape the RNA–RNA interaction spectrum, which influences phase separation. Taken together, m^6^A exhibits a huge potential to alter the conformation of m^6^A-harboring RNAs to affect their interaction with RBPs and RNAs, thereby modulating multivalence dynamics.

### Phase Separation Provides Platforms for m^6^A-Regulating Bioprocesses

**FIGURE 3:**
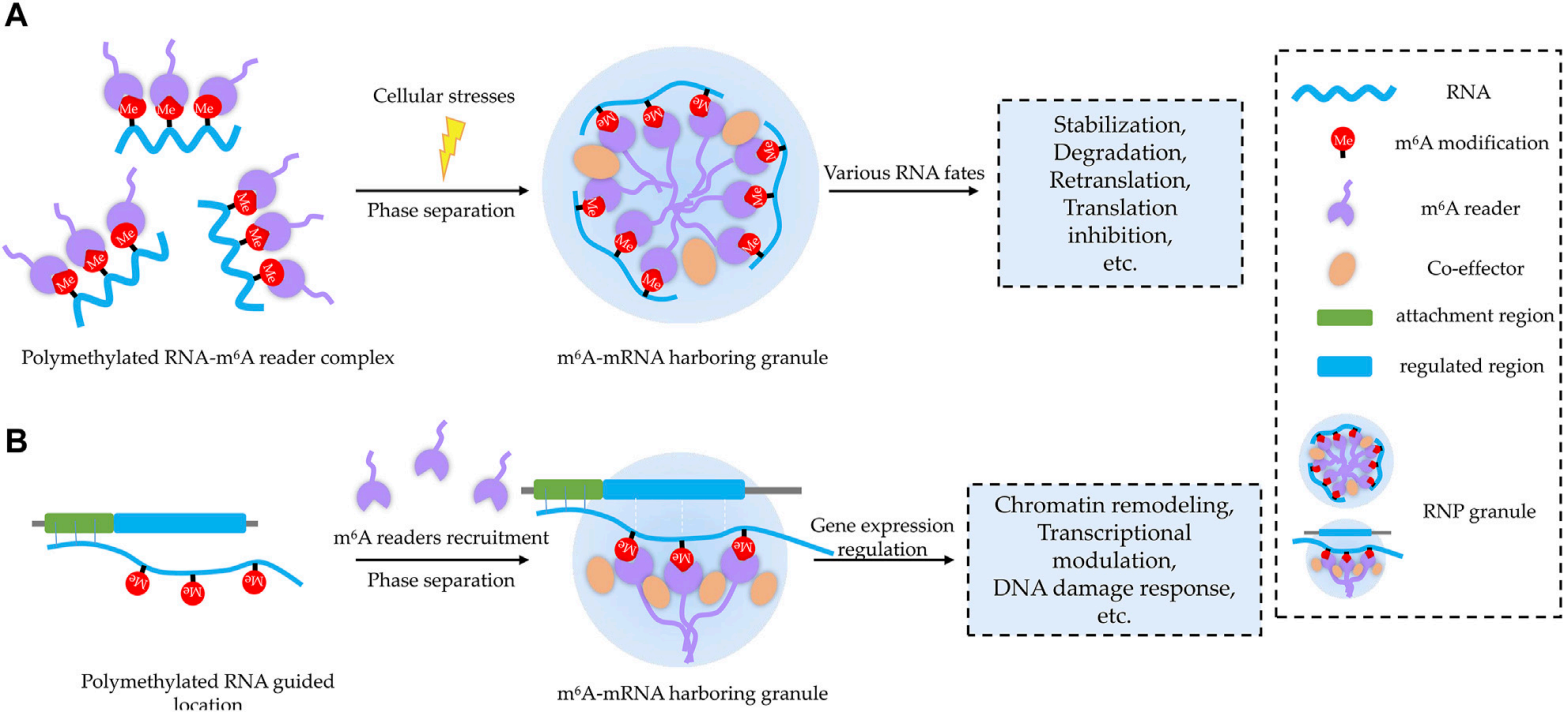
Two proposed working patterns for m^6^A-related BioMCs. **(A)** m^6^A modification acts as a sorting marker to decide RNA components and their associated molecular partners inside the condensates upon stress, and forming BioMCs will facilitate regulation of m^6^A RNA for stress response. **(B)** On nascent RNAs, m^6^A acts as a beacon to recruit m^6^A readers, and then m^6^A RNA–reader complexes stimulate formation of BioMCs, which play important roles for chromatin remodeling, transcriptional regulation, DNA damage response, etc.

Although m^6^A exhibits a great potential to modulate phase separation, the biological processes regulated through this manner remain largely unknown. Based on current literatures, we propose that phase separation may provide platforms for m^6^A-regulating bioprocesses in two major working patterns with potential biological significance: cellular stress response and gene expression regulation (Figure 3).

For the former pattern, RNA m^6^A modification may work as a sorting marker to decide RNA-targeting BioMCs (Figure 3A), as m^6^A-modified RNA is enriched in BioMCs in a valency- and stoichiometry-dependent manner (Ries et al., 2019; Wang et al., 2020), at least in terms of SGs and PBs. Although the molecular mechanism of sorting needs to be further elucidated, it may correlate with the greater potential of higher levels of modified RNA to achieve multivalent interactions. When cells are exposed to environmental stresses, the translation is inhibited and mRNAs dissociate from ribosomes; the increased cytosol pool of free mRNAs, especially the non-translating ones, binds to RBPs and initiates SGs coalescing (van Leeuwen and Rabouille, 2019; Mateju et al., 2020). As m^6^A-modified RNA–m^6^A reader complex is preferentially recruited to SGs, the readers can tune the fate of target mRNA with help from other associated co-effectors (Ries et al., 2019; Wang et al., 2015; Fu and Zhuang, 2020; Huang et al., 2018; Stöhr et al., 2006). For instance, YTHDF1 was regarded to promote translation re-initiation for its moderately high co-localization with eIF3/RPS6 in the periphery of G3BP1 cores in SGs (Wang et al., 2015), and the interaction of YTHDF1 with the translation machinery (RPS10) at the periphery of SGs was also reportedly essential for translation initiation promotion (Fu and Zhuang, 2020). Whereas, Ries et al. (2019) showed that YTHDFs’ role in phase separation is independent of their role in translation or degradation. Thus, YTHDF proteins seem to exert dual functions in protein translation and in the formation of SGs. Given the different conditions employed (thermal instead of oxidative stress) and different targets studied (overall mRNA instead of YTHDF target ones), it needs further investigation to reveal how YTHDF proteins discriminate the mRNAs that will be regulated at the translation level and the mRNAs that will be relocated into condensates. Interestingly, both the levels of m^6^A modification and m^6^A readers were increased under certain cellular stresses (Meyer et al., 2015; Zhou et al., 2015; Xiang et al., 2017; Anders et al., 2018; Maimaitiyiming et al., 2020), supporting the notion that m^6^A participates in stress response through modulating phase separation.

For the latter pattern, m^6^A occurring on nascent RNAs could act as a “beacon” to recruit m^6^A readers, which could further interact with other co-effectors to reshape the chromatin, regulate transcription, participate in DNA damage response, etc. (Figure 3B). For instance, the YTHDC1 anchoring in nascent RNA recruits H3K9me2 demethylase KDM3B to change the histone methylation levels of target chromatin (Li et al., 2020a). Similarly, SETDB1 and NCL-KAP1 are directed to the transcripts of retrotransposons by binding to YTHDC1 and deposit H3K9me3 in two cell state-related retrotransposons (Chen et al., 2021a; Liu et al., 2021). It is also evident from *XIST*-mediated X-chromosome inactivation. Several gene-silencing proteins may precisely localize on *XIST* by binding to YTHDC1 in an m^6^A-dependent manner (Patil et al., 2016). In addition to reshaping the chromatin, m^6^A could regulate transcription as well. For instance, YTHDC1 recognizes m^6^A sites in lncRNA *MALAT1*, which harbors multiple m^6^A motifs, playing an important role in reshaping nuclear speckles and modulating the accessibility of nuclear speckles to diverse genes, thereby affecting gene expression (Wang et al., 2021). In addition to *MALAT1*, another lncRNA, *NEAT1*, which is required for paraspeckle assembly through phase separation (Yamazaki et al., 2018), was also reported to regulate gene expression in an m^6^A-dependent manner (Wen et al., 2020).

Apart from transcriptional regulation, m^6^A in DNA damage-associated RNAs may also play an important role in DNA damage repair (Xiang et al., 2017; Zhang et al., 2020), and YTHDC1 anchoring in m^6^A sites of damage-associated RNA would facilitate the stabilization of DNA–RNA hybrids at damage sites and mediate the recruitment of RAD51 and BRCA1 for homologous recombination-mediated repair (Zhang et al., 2020). Interestingly, several lncRNAs (*XIST*, *MALAT1*, *NEAT1*, *LINE1*, dilncRNA, etc.) seem essential for nuclear phase separation events, which may be due to their length, flexible structure, and potentially multiple m^6^A sites. Multiple m^6^A-bearing mRNAs are predominantly located in the nucleoplasm and probably associated with chromatin remodeling in terms of molecular function in gene ontology (Ries et al., 2019).

RNA modifications other than m^6^A might also take part in phase separation since they are capable of changing the pattern of RNA–RNA and RNA–protein interactions as well (Lewis et al., 2017; Drino and Schaefer, 2018). Take N^1^-methyladenosine (m^1^A) for example, it occurs at the Watson–Crick interface and endows a positive charge to the modified adenosine, thereby changing RNA structure and RNA–protein interactions (Safra et al., 2017). A recent study reported that m^1^A is significantly accumulated in SGs upon heat shock and oxidative stress, along with its writer TRMT6/61A, likely hyposensitizing cells to the stress (Alriquet et al., 2021). This finding supports the notion that m^1^A modification participates in phase separation. More studies are needed in the future to figure out if phase separation is a universal mechanism to mediate modified RNAs’ sorting and to regulate their fate as well as function.

## Perspectives and Concluding Remarks

RNA m^6^A modification is an emerging layer of regulator over cellular BioMCs *via* phase separation (Liu et al., 2019a; Gao et al., 2019; Ries et al., 2019; Fu and Zhuang, 2020; Wang et al., 2020; Cheng et al., 2021; Lee et al., 2021). Under certain stress conditions, m^6^A modification acts as a sorting mark to enrich m^6^A-modified mRNAs in SGs and therefore potentially influence multiple cellular processes by modulating related mRNA re-translation after stress relief (Ries et al., 2019; Fu and Zhuang, 2020). These observations indicate the importance of m^6^A modification in stress-response mechanism and potentially in stress-related diseases. It has been reported that the arrest of BioMCs’ dynamics is correlated with some pathological processes (Mathieu et al., 2020). Therefore, an investigation of the details of m^6^A modification in phase separation would improve our understanding in stress response and related diseases.

Although specific RNAs harboring multivalent m^6^A modification have shown a strong potential to control cellular processes *via* phase separation, several technical problems limited the investigation of the biological function of m^6^A-mediated phase separation. First, a feasible approach to examine how an m^6^A-modified RNA regulates phase separation is condensate reconstitution experiment *in vitro* using artificially synthesized RNA and purified protein. However, it would be difficult to synthesize longer RNAs and add multiple m^6^A modifications in proper sites *in vitro* (Roden and Gladfelter, 2021). Second, the biomolecular condensates *in vivo* usually consist of numerous components including distinct RNA species and various RBPs, and it would be difficult to purify and include all components in an *in vitro* experiment. Third, the thermodynamic features of the heterotypic multicomponent interactions are different from *in vivo* condition and *in vitro*-purified components’ interaction in simplified model (Riback et al., 2020). Besides, unmodified RNA constitute a large portion of intracellular RNA and lots of m^6^A-modified mRNAs harbor only one m^6^A modification site (Dominissini et al., 2012; Zaccara et al., 2019); therefore, using multivalently modified RNA alone in reconstitute experiments may lead to false conclusions that deviate from physiological conditions.

Apart from *in vitro* experiments, the *in vivo* experiment is another available system to study how an m^6^A-modified RNA regulates phase separation. However, cellular BioMCs are usually constituted by multitudinous components, resulting in difficulty to evaluate the specific effects from m^6^A-modified RNAs. Additionally, traditional routes of studying m^6^A modification of a particular RNA often require defining the m^6^A site, changing the stoichiometry of m^6^A modification, and observing the consequent phenomenon. For this end, m^6^A regulators (writers, readers, and erasers) are often intervened (increase, decrease, mutate, etc.) to modulate m^6^A modification levels on target RNA or change the interaction pattern between target RNA and certain m^6^A readers of interest. However, these measures would lead to uncontrollable off-target effects, because these regulators are shared by thousands of RNAs apart from the targeted one. An improved method is to reconstruct the target RNA to change its modifiable capacity and the spectrum of binding partners; in many cases, it contains the deletion or mutation applied to the target RNA. Though this improved method greatly eliminates the off-target possibility and makes the intervention more precise, some other risks occur. As the ideal research objects are long RNAs harboring multiple m^6^A sites, such intervention may result in deletion or sequence component changes of large RNA fragments, both of which are important parameters for interaction spectrum contributing to RNA-mediated phase separation. Therefore, most of the currently available methods are more or less defective.

The ideal strategy is to site specifically modulate m^6^A levels and interaction spectrum of target RNA without changing the primary sequence. Recently, several biological tools have been developed to achieve site-specific m^6^A editing (Table 1) (Rauch et al., 2018; Liu et al., 2019b; Rauch et al., 2019; Li et al., 2020b; Mo et al., 2020; Shinoda et al., 2020; Wilson et al., 2020; Zhao et al., 2020; Chen et al., 2021b). The approach for developing new editing biology tools that interfere with RNA modification is using gRNA as the locator for the target sequence and refined Cas protein as the adaptor for the functional effectors to anchor and modulate target RNA. m^6^A writers, erasers, and readers were integrated with Cas protein to regulate m^6^A modification at specific sites on target RNA (Liu et al., 2019b; Rau et al., 2019; Li et al., 2020b; Mo et al., 2020; Chen et al., 2021b). For instance, Wilson et al. (2020) created nucleus-localized and cytoplasm-localized dCas13 fusions with a truncated METTL3 methyltransferase domain (nucleus-localized) and modified METTL3:METTL14 complex (cytoplasm-localized), which were able to install m^6^A in specific sites of target RNAs. Similarly, dCas13b fusions with ALKBH5 succeeded to specifically remove m^6^A of targeted mRNA. It is worth noting that one of the engineered tools exhibited equal efficiency in eliminating multiple modifications on a single target as well (Li et al., 2020b).

**TABLE 1:** Novel tools for site-specific m^6^A editing without primary sequence changed.

Category	Reconstituted construct	Working pattern	Ref
CRISPR–CAS-based	The fusion of YTHDF1 and dCas13b	SgRNA guides editing system to targeted transcript, and fusioned m^6^A readers function to achieve translation/degradation modulation	Rauch et al. (2018)
	The fusion of YTHDF2 and dCas13b		
	The fusion of M3M14 and dCas9	PAMer and sgRNA guide editing system to targeted site and fusioned m^6^A writer/eraser function to install/erase m^6^A modification	Liu et al. (2019b)
	The fusion of ALKBH5/FTO and dCas9		
	The fusion of ALKBH5 and dCas13b	SgRNA guides editing system to targeted transcript and fusioned ALKBH5 functions to erase m^6^A modification	Li et al. (2020b)
	The fusion of dCas13b and 10 copies of GCN4 peptides cooperates with scFv-fusion RNA demethylase	SgRNA guides dCas13b–GCN4 fusions to targeted transcript and further multiply recruit scFv fusion RNA demethylase to erase m^6^A modification	Mo et al. (2020)
	The fusion of METTL3/METTL3:METTL14 and dCas13	SgRNA guides editing system to targeted transcript and fusioned METTL3/METTL3:METTL14 function to achieve transmethylation	Wilson et al. (2020)
	The RNA anchor probes containing dCas13b and CIBN (a truncated version of light-sensitive protein CIB1) cooperate with the effector probe containing CRY2PHR(the photolyase homology region of CRY2) and METTL3/METTL14 or FTO	The RNA anchor binds the targeted RNA *via* crRNA, and METTL3/METTL14 or FTO is recruited as the attraction of CRY2PHR and CIBN heterodimerization upon blue light irradiation to install/erase m^6^A modification	Zhao et al. (2020)
	The fusion of dCas13a and ALKBH5	SgRNA guides editing system to targeted transcript, and fusioned ALKBH5 functions to erase m^6^A modification	Chen et al. (2021b)
CRISPR–CAS-inspired	The fusion of an effector protein, a RNA hairpin-binding protein, and ss-RNA-binding protein. YTHDF1/YTHDF2 was employed as effector protein, TBP/SLBP as RNA hairpin-binding protein, and ORF5/HBEGF/β-defensin as ss-RNA-binding protein	gRNA guide editing system to targeted site, RNA hairpin-binding protein binds to the structure of gRNA, ss-RNA-binding protein stabilizes and protects the gRNA prior to target engagement, and the effector protein works in a proximity-dependent manner	Rauch et al. (2019)
Others	The fusion of programmable RNA-binding protein PUF and METTL14	PUFs with specific mRNA-binding regions guide editing system to targeted transcript and fusioned METTL14/FTO function to install/erase m^6^A modification	Shinoda et al. (2020)
	The fusion of programmable RNA-binding protein PUF and FTO		

Another interesting and practical engineered tool is “TRADES” (Mo et al., 2020). Distinct from regular engineering practice, in which Cas protein serves as an adaptor to make the distance between the functional element fused to it and targeted RNA complementary with gRNA closer, in the TRADES system, the dCas13b portion is fused to 10 repeated GCN4 peptides, which are able to efficiently recruit multiple scFv–m^6^A eraser (scFv-FTO/ALKBH5) fusions. This design provides a wider editing window for its flexible repeated GCN4 peptides, and it would help to intervene with m^6^A clusters and eliminate m^6^A modification when the m^6^A sites are only known vaguely. Apart from CRISPR–Cas13 system, the PUF RNA-binding protein and CRISPR–Cas-inspired RNA targeting system (CIRTS) were also applied to regulate site-specific m^6^A (Rauch et al., 2019, Shinoda et al., 2020).

Taken together, we have summarized that RNA, as an essential portion of most BioMCs, can serve as drivers, regulators, and busters of BioMCs through modulating phase separation by multivalently interacting with biomacromolecules (protein and RNA). More importantly, RNA m^6^A modification, as the most widespread modification of eukaryotic mRNA, shows a strong potential to regulate phase separation and thus exert various physiological functions. Phase separation has been recognized as an emerging explanation for a plethora of previously unknown phenomena. Thus, it merits to comprehensively investigate how m^6^A regulates phase separation and how phase separation participates in m^6^A-mediated biological processes.
